# The conserved active site aspartate residue is required for the function of the chloroplast atypical kinase ABC1K1

**DOI:** 10.3389/fpls.2024.1491719

**Published:** 2024-11-19

**Authors:** Maud Turquand, Ana Rita Justo Da Silva, Thibaut Pralon, Fiamma Longoni, Felix Kessler, Joy Collombat

**Affiliations:** ^1^ Plant Physiology Laboratory, Institute of Biology, Université de Neuchâtel, Neuchâtel, Switzerland; ^2^ CDC-LAB, Plan-les-Ouates, Switzerland

**Keywords:** chloroplast, atypical kinase ABC1K1, photosynthesis, active site mutation, complementation

## Abstract

**Introduction:**

The Arabidopsis *abc1k1/pgr6* (Activity of BC1 complex/proton regulation 6) mutant is characterized by photosynthetic and conditional developmental phenotypes triggered by stressful red as well as high light. The Arabidopsis ABC1-like kinases belong to the atypical kinase family and contain conserved ATP-binding and hydrolysis motifs, but their physiological requirement has never been investigated.

**Methods:**

By mutation to asparagine, we demonstrate that the highly conserved active site aspartate residue within ATP-binding motif VIIb is required for the physiological functions of ABC1K1.

**Results:**

Complementation of the abc1k1 knock out mutant with ABC1K1 D400N, failed to restore the wildtype phenotype.

**Discussion:**

These results provide in vivo evidence for a critical role of the active site aspartate residue (D400) of ABC1K1.

## Introduction

**Figure 1:**
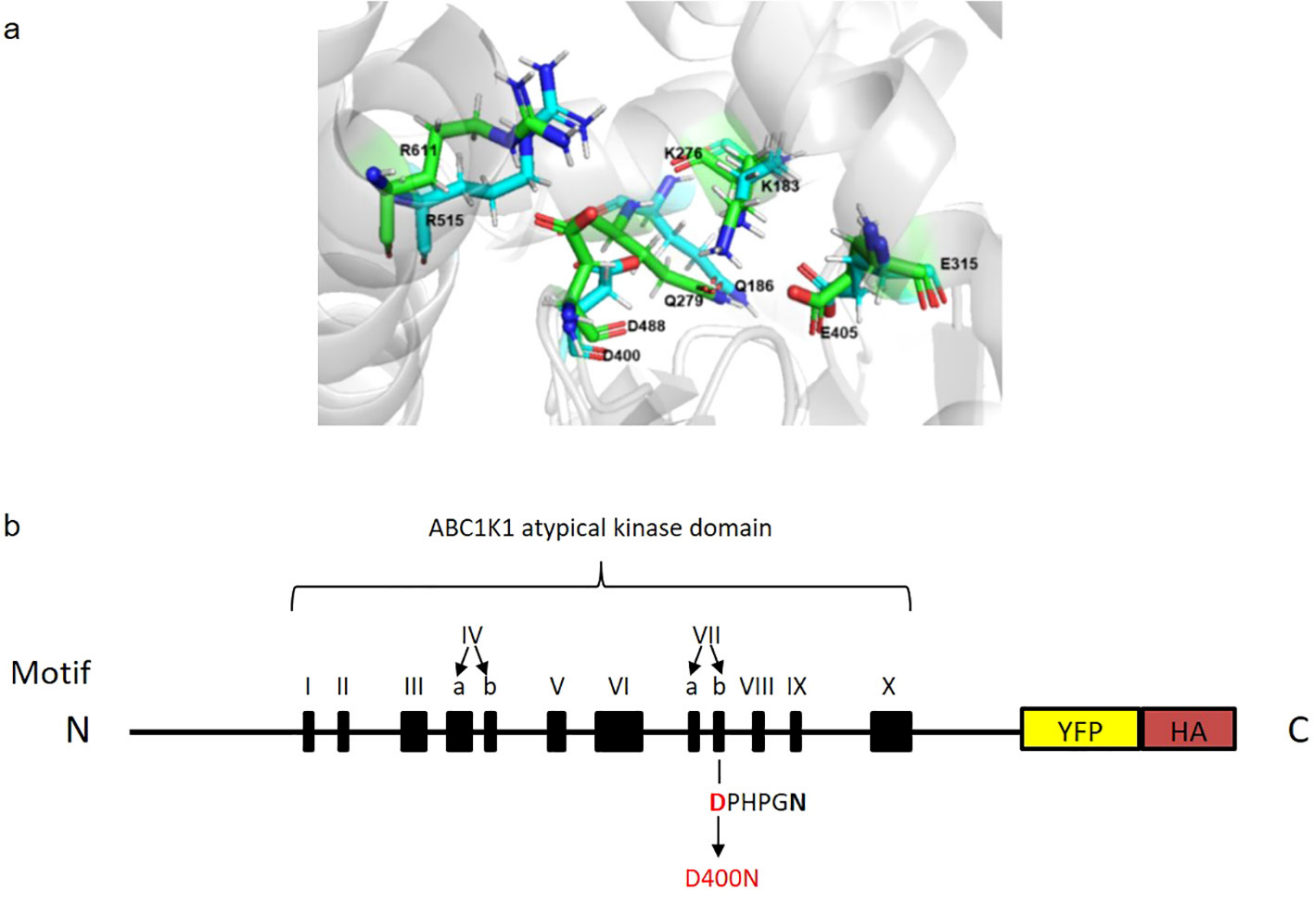
Predicted superimposed structures of ABC1K1 and COQ8A and schematic representation of recombinant ABC1K1-YFP-HA. **(A)** Representative snapshots of molecular dynamics (MD) simulations of COQ8A (PDB: 4PED) (green color) superimposed with ABC1K1(blue color) showing a precise alignment of the active site D400 in ABC1K1 and yeast COQ8A. **(B)** The recombinant ABC1K1 protein fused to a YFP-HA tag at the C-terminus was expressed under the control of 35S promoter. The black boxes indicate the positions of the 12 kinase motifs. The predicted atypical kinase active site corresponds to the highly conserved aspartic acid (D) at position 400 (in red) within motif VIIb (DPHPGN). D400 was mutated to asparagine (N) predicted to inactivate the kinase and/or ATPase activity of ABC1K1 (schematic representation of ABC1K1 motifs adapted from Lundquist et al., 2012).

Chloroplasts are green organelles dedicated to photosynthesis in eukaryotic photoautotrophic organisms. The chloroplast internal thylakoid membrane hosts the photosynthetic machinery necessary for the conversion of light energy into chemical energy. These membranes possess extrinsic spherical lipid microdomains called plastoglobules, the morphology and number of which vary with developmental stage and/or environmental conditions (Austin et al., 2006; Bréhélin et al., 2007; Zhang et al., 2010). Plastoglobules consist of a neutral lipid core enriched in prenyl lipids (such as tocopherols and plastoquinone) surrounded by a monolayer of polar lipids decorated with proteins (Eugeni Piller et al., 2012; Grennan, 2008; Kessler et al., 1999; Vidi et al., 2006). Among the around 30 proteins of the plastoglobule proteome, the most abundant are the fibrillins followed by the members of the ABC1 (Activity of BC1 complex) atypical kinase family (Grennan, 2008; Lundquist et al., 2012b; Ytterberg et al., 2006). ABC1Ks proteins are classified as atypical protein kinase (aPKs), which belong to the protein kinase-like (PKL) superfamily. They are evolutionarily conserved in archaea, bacteria and eukaryotes and contain a common ABC1 kinase domain of about 350 amino acids containing twelve conserved motifs five of which are also found in eukaryotic protein kinases (ePKs). These five highly conserved motifs are involved in ATP binding (motifs III, IVa and IVb), catalysis (motif VIIb) and Mg^2+^ chelation (motif VIII) (Lundquist et al., 2012a). Currently, there is no direct evidence of ABC1K mediated phosphorylation although some studies support the idea that it has protein kinase activity (Martinis et al., 2013; Xie et al., 2011). ABC1K homologs in yeast (ABC1/Coq8), *Escherichia coli* (YigR) and human (CABC1 or ADCK3) are all required for the biosynthesis of ubiquinone (Chang et al., 2018; Poon et al., 2000; Xie et al., 2011). Due to this, *Saccharomyces cerevisiae* ABC1/Coq8 is necessary for the activity of mitochondrial BC1 complex for cellular respiration and mutation of this protein leads to respiratory deficiency (Bousquet et al., 1991; Do et al., 2001). Similarly, mutations of the human ABC1 homologs were found in patients suffering of neurological disorders and cerebral seizures (Mollet et al., 2008). More recently, two additional ABC1K homologs, CQD1 and CQD2 have been shown to be implicated in ubiquinone subcellular distribution in *S. cerevisiae* (Kemmerer et al., 2021). In *Arabidopsis thaliana*, the ABC1K family has seventeen members localized in mitochondria or plastids, six of which have been found in plastoglobules (ABC1K 1, 3, 5,6, 7, 9) (Lundquist et al., 2012b; Ytterberg et al., 2006). Although the precise role of these ABC1K chloroplast proteins remains unclear, they are highly conserved across the plant kingdom and play an important role in chloroplast physiology and metabolism. For instance, ABC1K7 has been implicated in chloroplast lipid metabolism, iron distribution, oxidative stress response, and response to abscisic acid (Manara et al., 2016, 2015, 2014). The functions of ABC1K1 have been investigated while still not completely understood. The *abc1k1* mutant was first identified as *pgr6* (proton gradient regulation 6) mutant, characterized by high chlorophyll fluorescence, conditionally reduced NPQ and an impaired proton gradient (Martinis et al., 2014; Pralon et al., 2019; Shikanai et al., 1999). PSII efficiency, electron transport rate (ETR) and lipid composition are also strongly affected in this mutant particularly under high light (Lundquist et al., 2013; Martinis et al., 2014; Pralon et al., 2019). Pralon et al. showed that ABC1K1 is involved in maintaining the photoactive plastoquinone pool by regulating the plastoquinone distribution between plastoglobuli and the thylakoid membrane which may explain the mutant phenotype (Pralon et al., 2019). In addition to the photosynthetic role of ABC1K1, it has also been implicated in influencing plant development under specific light conditions. Under monochromatic red light, the *abc1k1* mutant has been identified as *bdr1* (bleached dwarf under red light), characterized by a short hypocotyl and very pale-green cotyledons and a strong reduction of phytochrome interacting factor (PIF) expression (PIF1, PIF3, PIF4 and PIF5) (Yang et al., 2016). In this study, we investigated the highly conserved, predicted active site aspartic acid residue D400 of ABC1K1, homologous to the predicted active site D488 of human CoQ8a/ADCK3 (Stefely et al., 2016) (**Figure 1A**). To do so, we complemented the *abc1k1* knockout mutant with a wildtype version of ABC1K1 (K1 WT, lines 1 and 2) fused to a HA-YFP tag or with a mutated version of ABC1K1, where D400 had been replaced by an asparagine (K1 D400N lines 1, 2 and 3). Here, we show that the D400 residue is required for proper function of ABC1K1 in photosynthesis regulation and in chloroplast biogenesis particularly under red and high light conditions.

## Results

### Isolation of *abc1k1* complemented lines and evaluation of ABC1K1 protein levels in different light conditions

To evaluate the importance of the predicted atypical kinase/ATPase domain of ABC1K1 protein for its function, we transformed an *abc1k1* T-DNA mutant (SALK_068628) with a construct expressing a C-terminally YFP-HA-tagged mutated version of ABC1K1, in which the highly conserved aspartic acid residue in the catalytic domain (Motif VIIb, position 400) had been replaced by an asparagine (*abc1k1* ABC1K1D400N-YFP-HA (abbreviated K1 D400N)) (**Figures 1A, B**). As a control, we complemented *abc1k1* with a C-terminally YFP-HA-tagged wildtype version of ABC1K1 (*abc1k1* ABC1K1-HA-YFP (abbreviated K1 WT)). Transformed plants were selected by segregation and verified by PCR, genotyping, and sequencing (**Supplementary Figure S1**).

**Figure 2:**
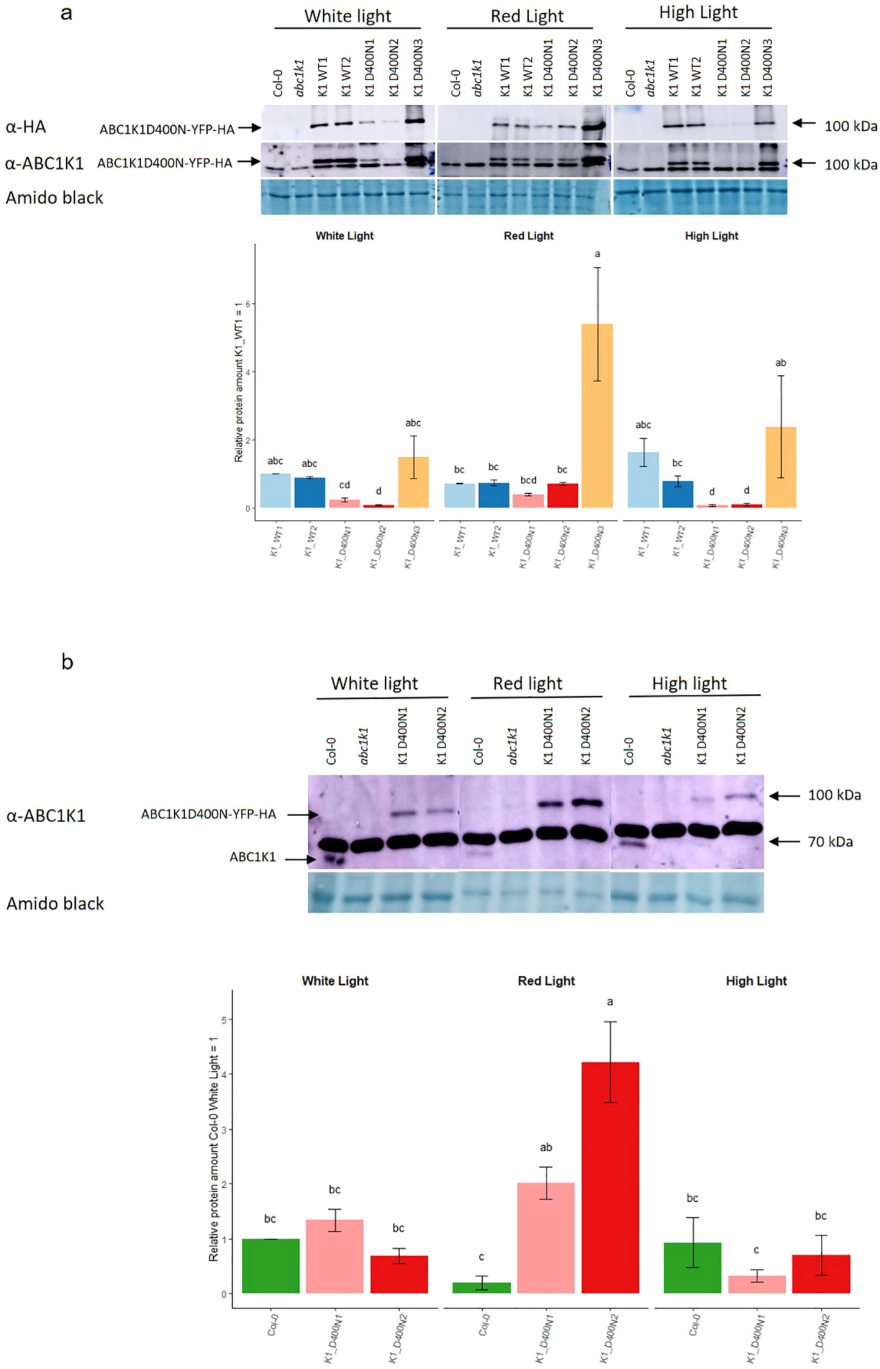
Expression of ABC1K1 proteins in complemented *abc1k1* lines under different light conditions. Total protein extracts from 5-day old seedlings grown under constant control white light (80 µE), red light (RL, 60 µE) or high light (HL, 500µE) and analyzed by western blot using an anti-HA or anti-ABC1K1 antibody. **(A)** Col-0, *abc1k1*, K1 WT1, K1 WT2, K1 D400N1, K1 D400N2 and K1 D400N3 were analyzed. The lower band observed with the anti-ABC1K1 antibody is non-specific. The histogram shows the average protein signal intensity of anti-HA. The histogram for anti-ABC1K1 is shown in **Supplementary Figure S2**. Error bars indicate the standard error between biological replicates (n=2). **(B)** Col-0, *abc1k1*, K1 D400N1 and K1 D400N2 analyzed. The strong band observed with the anti-ABC1K1 antibody is non-specific and same as in **(A)**. The histogram shows the average protein signal intensity of ABC1K1 compared to Col-0 under White light. Error bars indicate the standard error between biological replicates (n=3). The letters indicate statistically different groups obtained by *Post Hoc* analysis based on the marginal means (α<0.05).

The protein expression level was evaluated by SDS-PAGE followed by Western blotting (**Figure 2**). By using an anti-HA and anti-ABC1K1 antibody, we observed that the two K1 WT lines (K1 WT 1 and 2) and the third line of K1 D400N (K1 D400N 3) gave strong signals for recombinant ABC1K1 protein under control white light whereas those for the other two lines of K1 D400N (K1 D400N 1 and 2) were around 5 to 10 times weaker (**Figure 2A**, **Supplementary Figure S2**).

Interestingly, the amount of recombinant ABC1K1 protein varied depending on the light conditions. Under red light, the level of ABC1K1 in the two K1 WT lines (K1 WT1 and 2) lines decreased compared to control light while it increased in all K1 D400N lines (K1 D400N 1, 2 and 3) (**Figure 2A**).

To evaluate the levels of the endogenous ABC1K1 protein in Col-0, we used the anti-ABC1K1 antibody. In order to detect the weak signal of endogenous ABC1K1 protein in Col-0, we did western blotting experiments (**Figure 2B**), without the lines expressing the ABC1K1 protein the most (K1 WT1, K1 WT2 and K1D400N 3) that would obscure the signal of endogenous ABC1K1 in Col-0. We observed that under control light there was 1.3 times more ABC1K1D400N-YFP-HA in the K1 D400N1 line compared to endogenous ABC1K1 in Col-0 whereas K1 D400N2 expressed 1.4 times less (**Figure 2B**). Similar to what we previously observed for ABC1K1-YFP-HA in K1 WT 1 and 2 lines, endogenous ABC1K1 in Col-0 was decreased around 5 times under red light (**Figure 2B**) compared to control light conditions. The opposite, an increase in ABC1K1 levels under red light, was observed instead in K1 D400N1 and K1 D400N2 lines, having levels of around 1.5 and 6 times higher compared to control light (**Figure 2B**). Under high light, the level of ABC1K1 in Col-0 and ABC1K1D400N-YFP-HA in K1 D400N2 and K1 D400N1 remained constant or decreased slightly (**Figure 2B**).

### ABC1K1D400N does not complement the abc1k1 phenotypes occurring under high and red light

**Figure 3:**
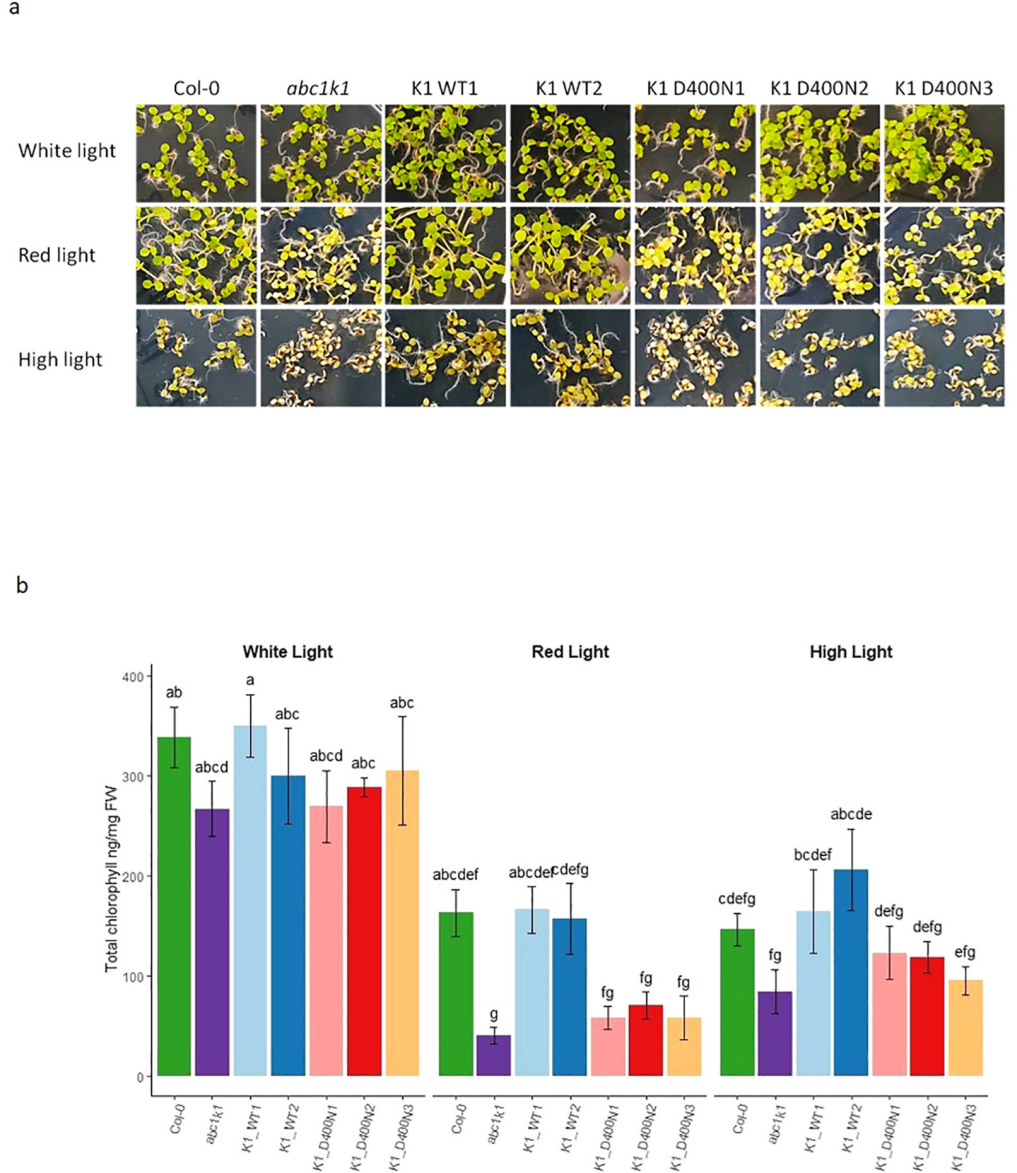
ABC1K1 D400N fails to restore the wildtype phenotype in *abc1k1.* **(A)** Phenotype and **(B)** Chlorophyll quantification of 5-day old Col-0, *abc1k1*, K1 WT1, K1 WT2, K1 D400N1, K1 D400N2 and K1 D400N3 grown on standard 0,5x MS media under constant control white light (CL, 80 µE), red light (RL, 60 µE) or high light (HL, 500µE). Error bars indicate the standard error between biological replicates (n=3). The letters indicate statistically different groups obtained by *Post Hoc* analysis based on the marginal means (α<0.05).

To determine whether the pale green phenotype of *abc1k1* under red and high light would be complemented by the wildtype or the mutated version of ABC1K1, plants were illuminated for 5 days with constant white light (80 µE), red light (680 nm, 60 µE), or high light (500 µE). In the two K1 WT1 and 2 lines the *abc1k1* phenotype under red and high light fully reverted to wildtype whereas the three K1 D400N lines maintained the *abc1k1* phenotype (**Figure 3A**). Chlorophyll quantifications of these lines confirmed full complementation in K1 WT 1 and 2 but not in K1 D400N 1, 2 and 3 (**Figure 3B**).

### Active site D400 is necessary for the ABC1K1 photosynthetic activity

**Figure 4:**
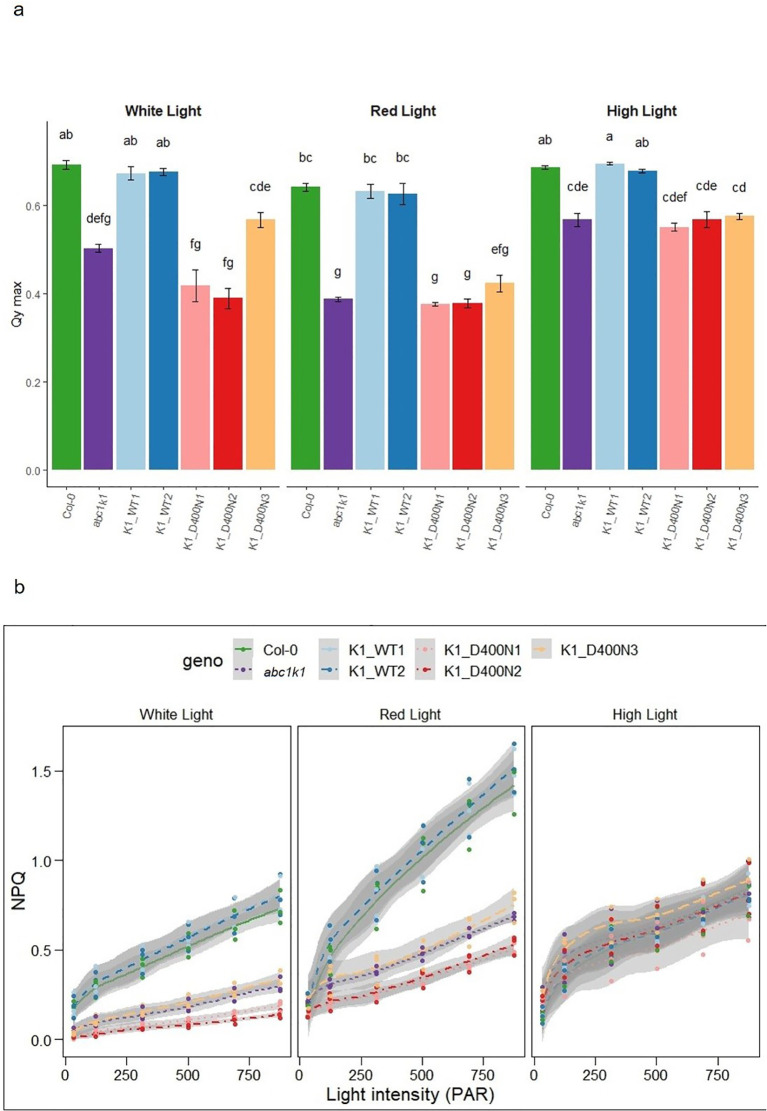
K1 D400N expressing lines display a photosynthetic defect similar to *abc1k1*. **(A)** PSII maximum quantum yield and **(B)** NPQ measured in 5-day old Col-0, *abc1k1*, K1 WT1, K1 WT2, K1 D400N1, K1 D400N2 and K1 D400N3 grown on standard 0,5x MS media under constant control white light (CL, 80 µE), red light (RL, 60 µE) or high light (HL, 500 µE). Error bars indicate the standard error between biological replicates (n=3). The letters in panel a indicate statistically different groups obtained by *Post Hoc* analysis (α<0.05).

*abc1k1* is characterized by a strong photosynthetic defect manifested in reduced PSII efficiency and non-photochemical quenching (NPQ) (Martinis et al., 2014; Pralon et al., 2019). To assess whether the predicted active site D400 of ABC1K1 is required for photosynthetic activity plants were grown under constant control white light (CL, 80 µE), red light (RL, 60 µE) or high light (HL, 500 µE). We determined the maximum quantum yield of PSII (φ _max_ (= F_V_/F_M_)), as well as NPQ using chlorophyll fluorescence analysis under increasing light intensity in all complemented lines. The K1 D400N1, 2 and 3 lines and the *abc1k1* mutant showed a very similar decrease of PSII maximum quantum yield compared to Col-0 regardless of the growth light conditions while K1 WT lines (1 and 2) fully complemented the defect (**Figure 4A**). PSII efficiency in *abc1k1* was already affected when grown under control light, whereas no visible phenotype was observed under this condition. Line K1 D400N 3, which expresses the most ABC1K1 protein, appeared to partially restore PSII efficiency, when grown under control light.

NPQ was strongly decreased in *abc1k1* and K1 D400N 1, 2 and 3 particularly when grown under control and red light while the NPQ values in K1 WT 1 and 2 were higher than in Col-0. When grown under high light, NPQ in Col-0 and K1 WT 1 and 2 was strongly diminished compared to plants grown under control or red light. NPQ in K1 D400N 1, 2 and 3 as well as *abc1k1* remained comparatively unchanged. The unexpected differences observed between red and high light may be due to differential developmental effects and increased zeaxanthin production under high light (Chauhan et al., 2023).

### The homeostasis of photosynthetic proteins depends on the effect of ABC1K1 on the D400 active site under red light

**Figure 5:**
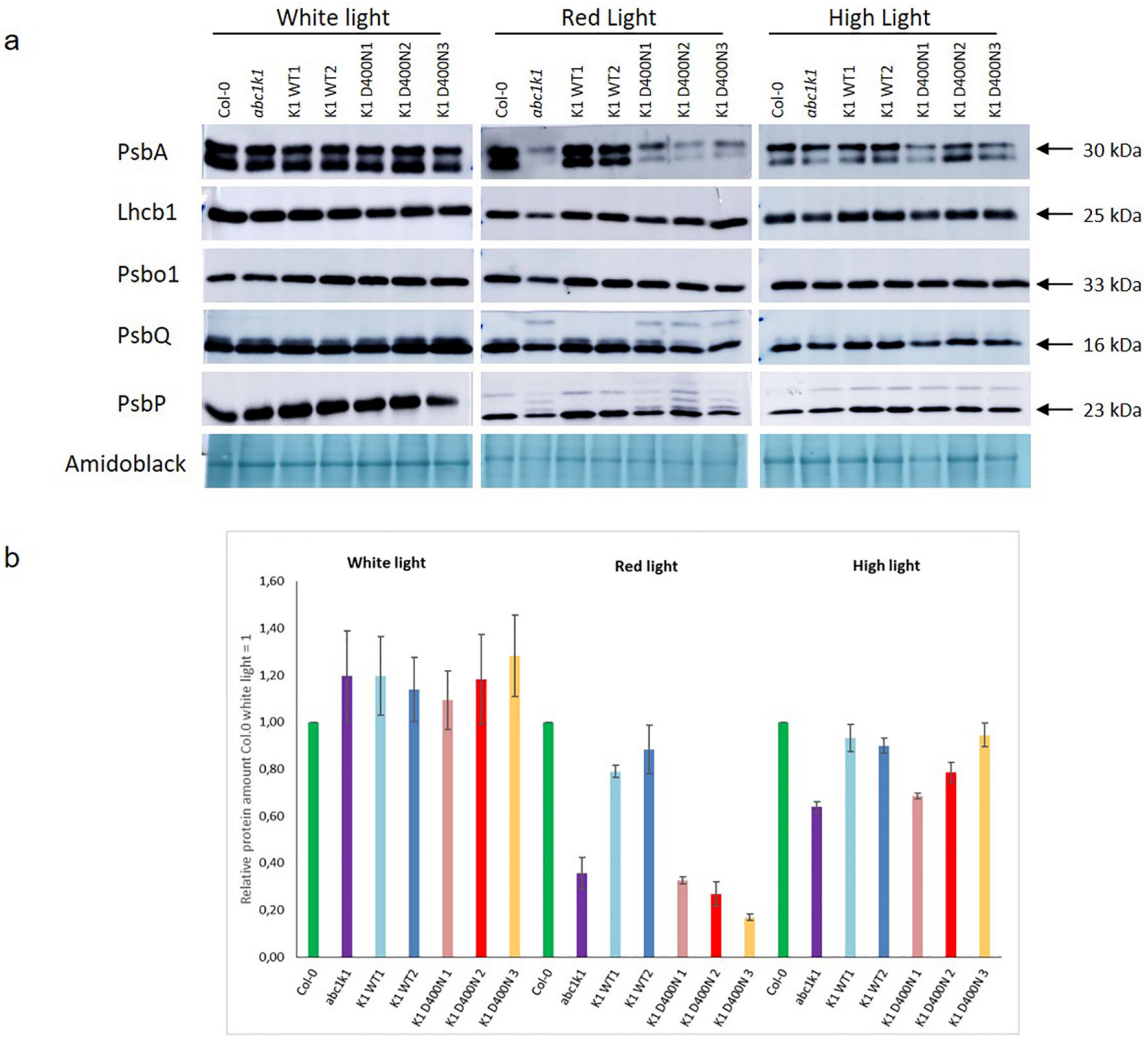
The levels of photosynthetic proteins are diminished in K1D400N expressing lines resembling *abc1k1* under red light. Total protein extract of 5-day old Col-0, *abc1k1*, K1 WT1, K1 WT2, K1 D400N1, K1 D400N2 and K1 D400N3 grown under constant control white light (CL, 80 µE), red light (RL, 60 µE) or high light (HL, 500µE) analyzed by western blot with an anti-PsbA, anti-Lhcb1, anti-Psbo1, anti PsbQ and anti-PsbP. **(A)** Amido black was used as loading control. The experiment was repeated on two biological replicates for PsbP proteins and three for all others. Col-0, *abc1k1*, K1 WT1, K1 WT2, K1 D400N1, K1 D400N2 and K1 D400N3 were analyzed. **(B)** The histogram show the average protein signal intensity of anti-PsbA compared to Col-0 under White light. The histograms for anti-Lhcb1, anti-PsbO1, anti-PsbQ and anti-PsbP are shown in **Supplementary Figure S3**. Error bars indicate the standard error between biological replicates (n=3).

ABC1k1 and the K1 D400N lines showed pale green phenotypes when grown under red or high light. In a separate study, we observed incompletely processed forms of thylakoid lumen proteins that accumulated in *abc1k1* under red light (including PsbP and PsbQ) while the mature forms of these protein as well as other photosynthesis-associated proteins were strongly down-regulated (Collombat et al., 2024). To determine whether this was also the case when the active site residue was mutated as in the K1 D400N lines, we analyzed the levels of PsbA, Lhcb1, PsbO1, PsbP and PsbQ in all complemented lines under control light, red light and high light by Western blot. Down regulation of photosynthesis-associated proteins in *abc1k1* was observed primarily under red light conditions (**Figure 5**), less so under high light and not at all control light (**Figure 5**). In the three K1 D400N lines the levels of PsbA, PspQ and PsbP under red light were not restored while in K1 WT 1 and 2 they were fully recovered. The presence of one or several additional bands of higher molecular mass for PsbQ and PsbP in *abc1k1* and in the three K1 D400N lines under red light was observed and may reflect effects on preprotein processing (Collombat et al., 2024).

## Discussion

ABC1K1 is a predicted atypical kinase involved in photosynthesis and red light mediated development (Martinis et al., 2014; Pralon et al., 2019; Yang et al., 2016). Young *abc1k1* mutant seedlings under red light are characterized by a pale green phenotype and are variegated after 14 days of high light (Pralon et al., 2019). So far, there is incomplete evidence for the molecular mechanisms standing behind these phenotypes, but perturbation of plastoquinone homeostasis appears to play a role (Pralon et al., 2019). Recently, we showed that preprotein processing of a small module of imported photosynthesis-associated proteins (PsbO1, PsbP1, PsbQ2, PsbT, PsaN, and PsaF) was impaired in *abc1k1* under red light and was associated with the failure of chloroplast biogenesis (Collombat et al., 2024).

In this study, we wished to determine whether the predicted atypical/ATPase domain of ABC1K1 is critical for its activity. For this purpose, the *abc1k1* knockout mutant was transformed with constructs expressing wildtype ABC1K1 WT or with ABC1K1D400N mutated at the kinase active site. ABC1K1 protein expression in all transformed lines reached at least the levels of endogenous ABC1K1 in the wildtype. The level of ABC1K1 expression in the K1 WT was close to the level found in line K1 D400N 3 lines under control light, which allows the direct comparison between these lines. Using the ABC1K1 antibody, we were able to compare the levels of transgenic ABC1K1 to the level of endogenous ABC1K1 in Col-0 under different light conditions (**Figure 2**). We observed that endogenous ABC1K1 in Col-0 under control light was hardly detectable by western blot presumably because its abundance is low and comparable to that of ABC1K1D400N in lines K1 D400N 1 and 2. In comparison, the ABC1K1 WT-expressing lines (K1 WT 1 and 2) as well as one ABC1K1D400N-expressing line (K1 D400N 3) had much higher levels of ABC1K1 than the Col-0 wild type.

Surprisingly, the level of ABC1K1 in Col-0 and the level of recombinant ABC1K1-WT in the K1 WT 1 and 2 lines decreased under red light compared to control light while ABC1K1D400N increased in K1 D400N lines. These results suggests that red light destabilizes the ABC1K1 protein and that its stability may be affected by its catalytic domain. This may be due to autophosphorylation a property that has been reported for the human Coq8a/ADCK3 homolog (Stefely et al., 2016).

ABC1K1D400N did not complement the *abc1k1* phenotype observed under either red or high light conditions which was reflected by the low chlorophyll levels in K1 D400N lines which were fully restored in K1 WT lines (**Figure 3**). The atypical kinase domain of ABC1K1 also seemed to be required for full photosynthetic activity since K1 D400N lines did not complement the PSII deficiency and NPQ defects characteristic of the *abc1k1* mutant grown under control, red and high light (**Figure 4**). Interestingly, these two photosynthetic parameters are already negatively affected when grown under control light in *abc1k1* and K1 D400N lines while no phenotype was observed under this condition. This observation may be attributed to the perturbation of plastoquinone homeostasis that allows normal growth under control light but affects chlorophyll fluorescence measurements under increasing light intensities. Finally, protein analysis showed that *abc1k1* and K1 D400N lines have similarly reduced levels of PsbA, PsbQ and PsbP whereas K1 WT 1 and 2-lines were like the Col-0 wildtype grown under red light (**Figure 5**).

As the protein levels of ABC1K1D400N in K1 D400N were at least as high as the endogenous protein in Col-0 wildtype (**Figures 2A, B**), its failure to complement the *abc1k1* phenotype is most likely due to the D400N mutation. The *abc1k1* phenotype is associated with strong downregulation of photosynthesis-associated proteins and accumulation of partially processed preproteins but this was only observed after growth under red light but not under the high light condition (**Figure 3**). This suggested that defects in *abc1k1* as well as K1 D400N are exacerbated specifically under Photosystem II-specific red light, a condition which unbalances excitation energy between Photosystems II and -I and primarily damages Photosystem II. Although photosynthetic defects can be measured in *abc1k1* and K1 D400N lines grown under control light, the photosynthesis-associated protein levels were not affected.

In summary, the findings obtained with the D400N mutant indicate that the conserved active site aspartate residue D400 of ABC1K1 is required for development and photosynthesis which is particularly evident when grown under red light (**Figure 4**).

## Materials and methods

### Plant materials, growth conditions and treatments

*Arabidopsis thaliana* wild-type refers to var. Columbia-0 (Col-0). *abc1k1* mutant is a T-DNA insertion line (SALK_068628) obtained from the Nottingham Arabidopsis Stock Centre (NASC, http://arabidopsis.info). The *abc1k1* mutant was complemented with 35S:ABC1K1-YFP-HA or 35S:ABC1K1D400N-YFP-HA. The pEarlyGate101-ABC1K1 plasmid was created by introducing the ABC1K1 coding sequence between the 35S promoter and the YFP-HA tag of pEarleyGate101 plant expression vector (Earley et al., 2006) by GeneCust’s (Boynes, France). The resulting plasmid was used to transform *Agrobacterium tumefaciens* C58 strain by electroporation. The modified strain was used to transform *abc1k1* mutant plants using the Floral Dip method (Clough and Bent, 1998). Seeds from transgenic plants were harvested and the mutation selected by resistance to 30 mg.l-1 glufosinate ammonium in ½ MS medium. Segregation analysis was performed to obtain homozygous 35S:ABC1K1-YFP-HA and 35S:ABC1K1D400N-YFP-HA lines with a single transgene insertion. Protein expression was confirmed by immunoblot in selected complemented plants. The next generation of seeds was sterilized and spread on 0.5x MS plates then placed in the dark for 24 hours at 4°C.Seeds were moved to 22-24°C and exposed for 1 hour to white light (80 µmol m−2 s−1), afterwards were kept for 5 days under continuous white light (80 µmol m−2 s−1) or moved to continuous red light (60 µmol m−2 s−1) or high light (500 µmol m−2 s−1). 5-day old seedlings were then collected under the light, immediately frozen in liquid nitrogen and stored at -20°C.

### Cloning, production and purification of ABC1K1 protein for antibody production

#### Cloning of ABC1K1 gene into pet21D vector

The ABC1K1 gene without transit peptide was amplified by PCR from cDNA. The amplified fragment was cloned into a PET21d vector using the Gibson Assembly Cloning Kit. Then, DH5α bacteria were transformed with the ligation product: 5 µl of the cloning product were added to 100 µl of DH5α and put on ice for 30 minutes. Then, the tube was put at 42°C for 40 seconds and replaced on ice for 5 minutes. 800 µl of LB were added to 100 µl of transformed DH5α and bacteria were regenerated for 1 hour at 37°C with agitation. Finally, DH5α were centrifuged 1 minutes at 16,000 g and concentrated into 100 µl of LB to be spread on solid LB medium supplemented with ampicillin. All the growing colonies were tested by PCR using primers T7-pet21d-Fw and T7-term-Rev. (a colony containing the pET21D plasmid with the ABC1K1 gene was selected, isolated and used to inoculate 3 ml of LB + ampicillin culture incubated for 24 hours at 37°C). Plasmids were finally extracted and purified using the miniprep system (Zymo Research).

#### Production of ABC1K1 protein

After checking the pET21D/ABC1K1 plasmid by sequencing, it was transformed into 100 µl of BL21(DE3) bacteria using the same protocol described above to allow the production of the ABC1K1 protein. 3 ml of LB + ampicillin was inoculated with a BL21(DE3) colony containing the pET21d/ABC1K1 plasmid and incubated at 37°C for 24 hours. Then, the 3 ml preculture was used to inoculate 50 ml of LB + ampicillin and incubated for 24 hours at 37°C. Finally, 6 l of LB + ampicillin were prepared using the 50 ml culture and placed at 37°C with gently agitation. When the DO of the culture reached 0.6, 0.5 mM IPTG was added and the culture incubated at 28°C overnight (16 hours) to allow ABC1K1 protein production and avoid degradation. After 16 hours, culture was centrifuged 20 minutes at 4,500 g to pellet the bacteria.

#### Purification of the ABC1K1 protein

The bacteria pellet was resuspended in 30 ml (4 ml.g-1 of pellet) of resuspension buffer containing (50 mM Tris-HCL pH8, 300 mM NaCl, 10 mM imidazol). 1 mg.ml-1 of lysozyme (Roche) was added to allow the cell lysis and the solution was put on a rotating device for 30 minutes at room temperature. The bacteria were then broken by high pressure using a French press system and treated with DNAse (Roche), (10 U.ml-1) to remove the DNA. Then, ultracentrifugation for 1 hour at 40,000 g allowed to separate soluble proteins from insoluble ones and cellular debris. After analyses of the supernatant and the pellet by western blot using an anti-HA antibody, we observed that most of the ABC1K1 protein was insoluble and stayed in the pellet. Therefore, the pellet was resuspended into 10 ml of resuspension buffer and 8 M of urea was added to solubilize the ABC1K1 protein. The solution was then dialyzed into 3 l of resuspension buffer for 3 hours and then 16 hours in fresh solution to remove urea. After the dialysis and the removal of the urea, a pellet was formed in the dialysis bag containing most of the ABC1K1 protein. This pellet was resuspended in 3 ml of PBS buffer + SB buffer (2% SDS, 5% β-mercaptoethanol, 10% glycerin, 0.1% Bromophenol Blue) and denatured for 20 minutes at 99°C. The solution was then loaded into a big 10% acrylamide gel to separate the proteins. A Coomassie coloration of the gel allowed the staining of proteins and the extraction of the gel portion corresponding to the size of ABC1K1. To remove the ABC1K1 protein from the gel, the gel portion was introduced into a dialysis bag with 2 ml of running buffer (125 mM Tris-HCL pH8.3, 1.25 M glycine, 0.5% SDS) and placed in an electrophoresis system containing 1 l of running buffer. Migration of the ABC1K1 protein from the gel to the running buffer solution was performed for 4 hours at 100 V. After the migration, the gel portion was eliminated and the 2 ml solution of running buffer was collected and tested by western blot to check the presence of the ABC1K1 protein.

#### Preparation of the ABC1K1 protein for antibody production

Dialysis of the 2 ml of running buffer solution containing the ABC1K1 protein was performed in 100 ml of PBS buffer 1X (3 hours and then overnight in fresh PBS). A last verification of the presence of the ABC1K1 in the PBS solution was performed after the dialysis by western blot. Samples were then lyophilized using the speedvac system and sent to Eurogentec for immunization, Belgium.

### Chlorophyll quantification

Total chlorophyll was extracted from 5-day old seedlings (minimum of 20 mg of fresh weight) by adding 10 µl per mg FW of DMF (Dimethylformamide). Samples were centrifuged 1 minute at 16,000 g and kept overnight at 4°C in the dark. Extracts were once again centrifuged for 3 minutes at 16,000 g before measuring the absorbance at 664 nm and 647 nm with a Nanodrop spectrophotometer (NanoDrop ND-1000 413 spectrophotometer, Witec AG). Total chlorophyll concentrations were calculated according to (Porra et al., 1989).

### Photosynthetic parameters

Maximum quantum yield of PSII (Φmax) and NPQ were measured using a Fluorcam MF800 (Photon System Instrument, Czech Republic, http://www.psi.cz). The actinic light was provided by blue LED (470 nm). The protocol starts by the measurement of the Φmax = (FM–FO)/FM where FM is the maximal fluorescence in dark acclimated plants, measured during a saturating light pulse, and FO the fluorescence in the dark. The actinic light intensity was increased by 1 minute steps. At the end of each light intensity step we determined the non-photochemical quenching NPQ = (FM – FM’)/FM’. FM’ is the maximal fluorescence at the end of each light intensity step. The experiment was performed on three independent biological replicates composed of 10 to 30, 5-day old seedlings per genotype.

### Immunoblot analyses

Frozen 5-day old seedlings were ground in 400 µl of lysis buffer (100 mM Tris-HCl pH8.5, 2% SDS, 10 mM NaF, 0.05% of protease inhibitor cocktail for plants (Sigma)) with a micro-pestle in a 1.7 ml microtubes. Samples were heated at 37°C for 30 minutes and centrifuged for 15 minutes at 16,000 g at room temperature. Protein concentration in each sample was determined using the Pierce BCA protein assay kit (Thermo Scientific, cat. No. 23225) following manufacturer instructions. Proteins were precipitated by chloroform-methanol, then resuspended in sample buffer (50 mM Tris-HCl pH6.8, 100 mM Dithiothreitol, 2% SDS, 0.1% Bromophenol Blue, 10% Glycerol) at a final concentration of 1 µg.µl-1 and denatured for 10 minutes at 65°C. 5 µl was loaded on a 16% polyacrylamide SDS gel and proteins were separated by electrophoresis before transfer to a nitrocellulose membrane for immunoanalysis using the following antibodies: anti-HA (Agrisera AS15 2924), anti-PsbA (Agrisera AS05 084) anti-Lhcb1 (Agrisera, AS01 004), Anti-PsbO1 (Agrisera AS14 2824), Anti-PsbP (Agrisera AS06 142-23), anti-PsbQ (Agrisera AS06 142-16). Primary antibodies were decorated with horseradish peroxidase-conjugated anti-rabbit (Merck, AP132P) or anti-mouse (Sigma, A5278) antibodies. Chemiluminescent signals were detected using a home-made reaction mixture (luminol 1.25 mM, cumaric acid 0.20 mM, mixed with 0.01% H_2_O_2_ just before the reaction) using a CCD camera (Amersham Imager 600, AmershamBiosciences, Inc.). The quantification of protein signals was done with the ImageQuant TL software and the quantification of amido black coloration was performed using ImageJ software.

#### Statistical analyses

The normal distribution of the residuals of each data set was tested before the statistical analysis. The data were analyzed with a Kruskal–Wallis rank sum test. If the result was significant and a linear model fitted the data, we then used the *post hoc* test for multiple comparisons with Šidák’s p-value correction. The calculated p-values were used to define statistically different groups with an alpha of 0.05. For the remaining data the comparison was made using the *Post-hoc* test of the Kruskal Agricolae package using the Bonferroni correction of the p values. The calculations were performed with RStudio (Version 2023.09.1 Build 494 RStudio Inc).

## Data Availability

The original contributions presented in the study are included in the article/**Supplementary Material**. Further inquiries can be directed to the corresponding author.

## Supplementary material

Supplementary Figure 1 Isolation and validation of complemented *abc1k1* lines. **(A)** Verification of the *abc1k1* knock out by PCR using primers *abc1k1*-1_RP, *abc1k1*-1_LP and LBb1.3 listed in **Supplementary Table S1**. **(B)** Verification of the presence of recombinant abc1k1 gene using 35S_pcL60 and YFP_R primer listed in **Supplementary Table S1** and sequencing results confirming the absence of the catalytic aspartic acid in position 400 of the protein in K1 D400N1, K1 D400N2 and K1 D400N3.

Supplementary Figure 2 Average protein signal intensity of anti-ABC1K1 Total protein extract from 5-day old Col-0, *abc1k1*, K1 WT1, K1 WT2, K1 D400N1, K1 D400N2 and K1 D400N3 grown under constant control white light (80 µE), red light (RL, 60 µE) or high light (HL, 500 µE) analyzed by western blot using an anti-ABC1K1 antibody. The histogram shows the average protein signal intensity of ABC1K1 compare to Col-0 under White light. Error bars indicate the standard error between biological replicates (n=2). The letters indicate statistically different group obtained by *Post Hoc* analysis based on the marginal means (α<0.05).

Supplementary Figure 3 Average protein signal intensity for various antibodies Total protein extract from 5-day old Col-0, *abc1k1*, K1 WT1, K1 WT2, K1 D400N1, K1 D400N2 and K1 D400N3 grown under constant control white light (CL, 80 µE), red light (RL, 60 µE) or high light (HL, 500µE) analyzed by western blot with an anti-HA, anti-PsbA, anti-Lhcb1, anti-Psbo1, anti PsbQ and anti-PsbP. The histograms show the average protein signal intensity of different proteins compared to Col-0 under White light. Error bars indicate the standard error between biological replicates (n=2 for anti-PsbP or n=3 for others).

Supplementary Table 1 Primers used for genotyping and sequencing of *abc1k1 c*omplemented lines The abc1k1 t-DNA insertion has been verified in all complemented lines by PCR using abc1k1-1_RP, abc1k1-1_LP and LBb1.3 primers. The presence of the ABC1K1 recombinant gene has been checked by PCR using 35S_pcL60 and YFP_R primer. Finally, the sequencing of the amplified fragment with T7-pet21d-Fw and T7-term-Rev primers confirmed the presence of the mutation in K1 D400N lines and its absence in K1 WT lines.
