# Supplementary figures and images for: The conserved active site aspartate residue is required for the function of the chloroplast atypical kinase ABC1K1
Source: Front Plant Sci. 2024 Nov 19;15:1491719. doi: 10.3389/fpls.2024.1491719 (PMC11613423; doi:10.3389/fpls.2024.1491719)

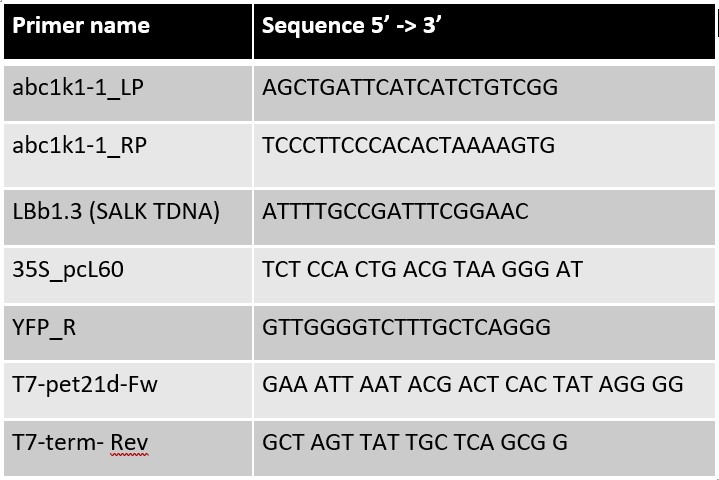

Supplement: Supplementary file 1 [file Image4.jpeg]

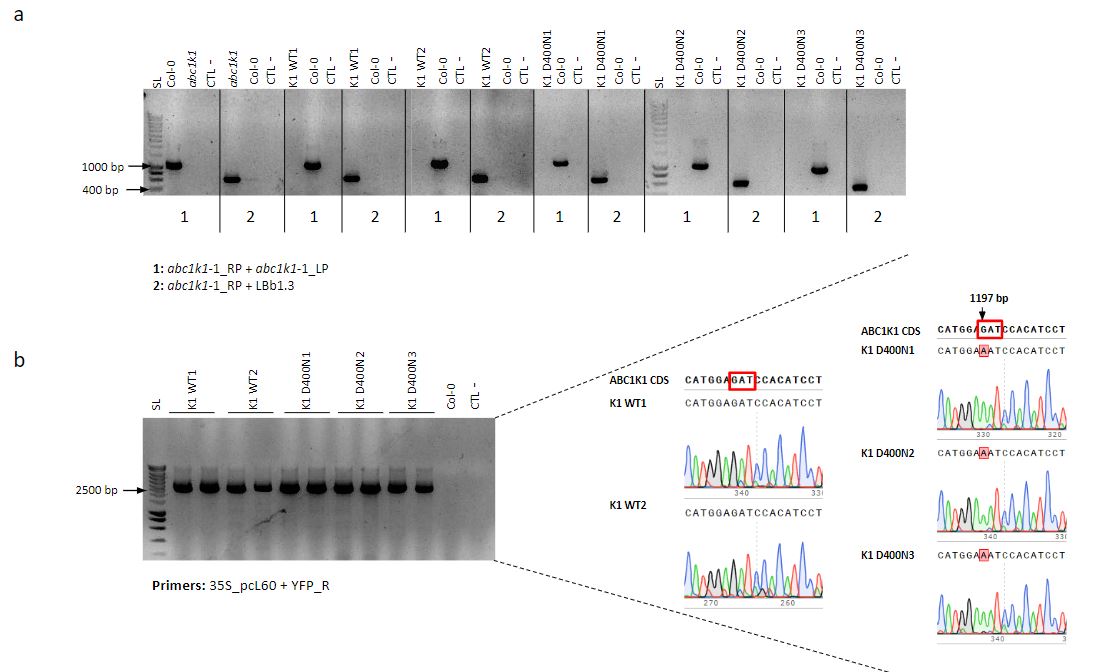

Supplement: Supplementary Figure 1 — Isolation and validation of complemented abc1k1 lines. (A) Verification of the abc1k1 knock out by PCR using primers abc1k1-1_RP, abc1k1-1_LP and LBb1.3 listed in Supplementary Table S1 . (B) Verification of the presence of recombinant abc1k1 gene using 35S_pcL60 and YFP_R primer listed in Supplementary Table S1 and sequencing results confirming the absence of the catalytic aspartic acid in position 400 of the protein in K1 D400N1, K1 D400N2 and K1 D400N3. [file Image1.jpeg]

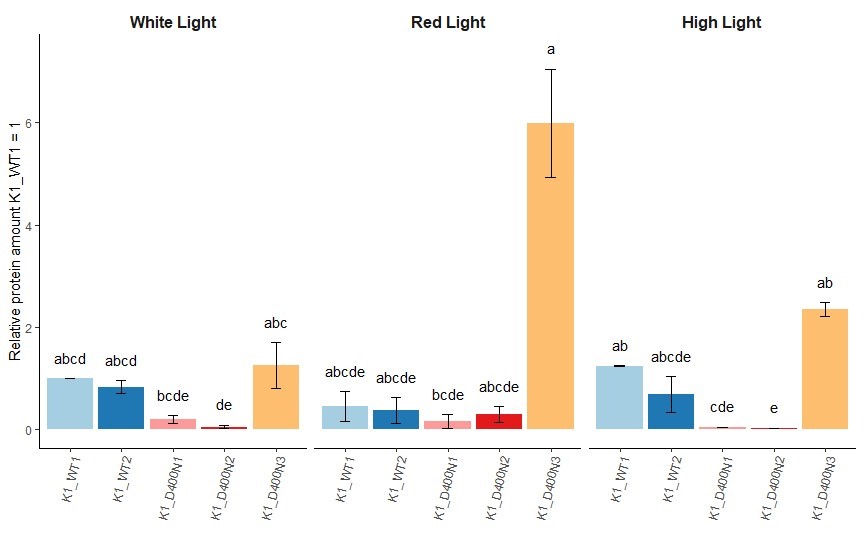

Supplement: Supplementary Figure 2 — Average protein signal intensity of anti-ABC1K1 Total protein extract from 5-day old Col-0, abc1k1, K1 WT1, K1 WT2, K1 D400N1, K1 D400N2 and K1 D400N3 grown under constant control white light (80 µE), red light (RL, 60 µE) or high light (HL, 500 µE) analyzed by western blot using an anti-ABC1K1 antibody. The histogram shows the average protein signal intensity of ABC1K1 compare to Col-0 under White light. Error bars indicate the standard error between biological replicates (n=2). The letters indicate statistically different group obtained by Post Hoc analysis based on the marginal means (α<0.05). [file Image2.jpeg]

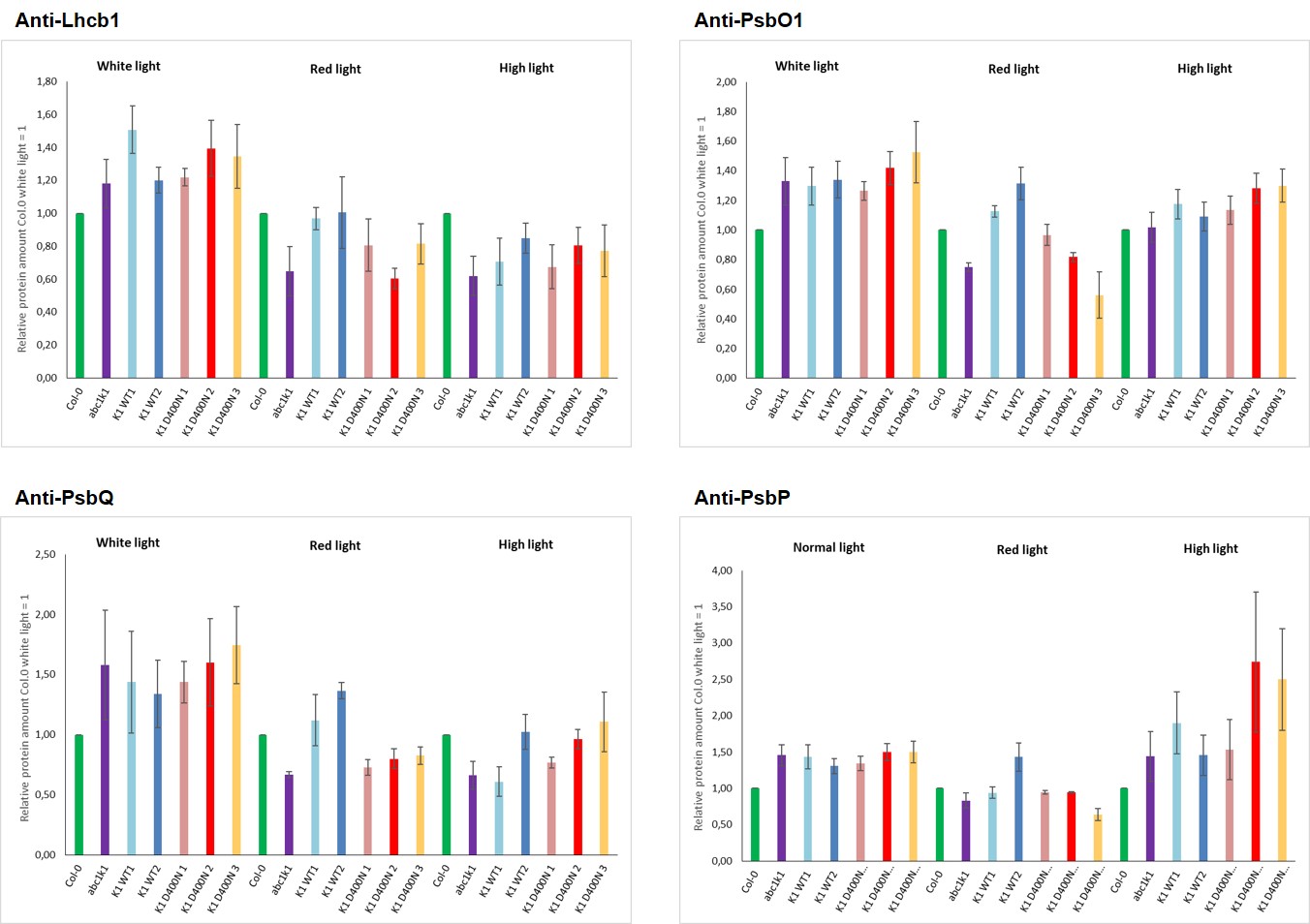

Supplement: Supplementary Figure 3 — Average protein signal intensity for various antibodies Total protein extract from 5-day old Col-0, abc1k1, K1 WT1, K1 WT2, K1 D400N1, K1 D400N2 and K1 D400N3 grown under constant control white light (CL, 80 µE), red light (RL, 60 µE) or high light (HL, 500µE) analyzed by western blot with an anti-HA, anti-PsbA, anti-Lhcb1, anti-Psbo1, anti PsbQ and anti-PsbP. The histograms show the average protein signal intensity of different proteins compared to Col-0 under White light. Error bars indicate the standard error between biological replicates (n=2 for anti-PsbP or n=3 for others). [file Image3.jpeg]
